# Molecular Landscape of LncRNAs in Prostate Cancer: A focus on pathways and therapeutic targets for intervention

**DOI:** 10.1186/s13046-022-02406-1

**Published:** 2022-07-01

**Authors:** Sepideh Mirzaei, Mahshid Deldar Abad Paskeh, Elena Okina, Mohammad Hossein Gholami, Kiavash Hushmandi, Mehrdad Hashemi, Azuma Kalu, Ali Zarrabi, Noushin Nabavi, Navid Rabiee, Esmaeel Sharifi, Hassan Karimi-Maleh, Milad Ashrafizadeh, Alan Prem Kumar, Yuzhuo Wang

**Affiliations:** 1 Department of Biology, Faculty of Science, Islamic Azad University, Science and Research Branch, Tehran, Iran; 2 Department of Genetics, Faculty of Advanced Science and Technology, Tehran Medical Sciences, Islamic Azad University, Tehran, Iran; 3 Farhikhtegan Medical Convergence sciences Research Center, Farhikhtegan Hospital Tehran Medical Sciences, Islamic Azad University, Tehran, Iran; 4 Department of Pharmacology, Yong Loo Lin School of Medicine, National University of Singapore, Singapore, 117600 Singapore; 5 NUS Centre for Cancer Research (N2CR), Yong Loo Lin School of Medicine, National University of Singapore, 180554 Singapore, Singapore; 6 Faculty of Veterinary Medicine, Kazerun Branch, Islamic Azad University, Kazerun, Iran; 7 Department of Food Hygiene and Quality Control, Division of epidemiology & Zoonoses, Faculty of Veterinary Medicine, University of Tehran, Tehran, Iran; 8 School of Life, Health & Chemical Sciences, The Open University, Milton Keynes, United Kingdom; 9 Pathology, Sheffield Teaching Hospital, Sheffield, United Kingdom; 10 Department of Biomedical Engineering, Faculty of Engineering and Natural Sciences, Istinye University, 34396 Istanbul, Turkey; 11 Department of Urologic Sciences and Vancouver Prostate Centre, University of British Columbia, V6H3Z6, Vancouver, BC Canada; 12 Department of Materials Science and Engineering, Pohang University of Science and Technology (POSTECH), 77 Cheongam-ro, Nam-gu, Pohang, Gyeongbuk 37673 Korea; 13 School of Engineering, Macquarie University, Sydney, New South Wales 2109 Australia; 14 Department of Tissue Engineering and Biomaterials, School of Advanced Medical Sciences and Technologies, Hamadan University of Medical Sciences, Hamadan, 6517838736 Iran; 15 School of Resources and Environment, University of Electronic Science and Technology of China, P.O. Box 611731, Xiyuan Ave, Chengdu, PR China; 16 Department of Chemical Engineering, Quchan University of Technology, Quchan, Iran; 17 Department of Chemical Sciences, University of Johannesburg, Doornfontein Campus, Johannesburg, 2028 South Africa; 18 Faculty of Engineering and Natural Sciences, Sabanci University, Orta Mahalle, Üniversite Caddesi No. 27, Orhanlı, Tuzla, 34956 Istanbul, Turkey

**Keywords:** Prostate cancer, Long non-coding RNA (lncRNA), MicroRNA, Drug resistance, Immune evasion, Exosome

## Abstract

**Background:**

One of the most malignant tumors in men is prostate cancer that is still incurable due to its heterogenous and progressive natures. Genetic and epigenetic changes play significant roles in its development. The RNA molecules with more than 200 nucleotides in length are known as lncRNAs and these epigenetic factors do not encode protein. They regulate gene expression at transcriptional, post-transcriptional and epigenetic levels. LncRNAs play vital biological functions in cells and in pathological events, hence their expression undergoes dysregulation.

**Aim of review:**

The role of epigenetic alterations in prostate cancer development are emphasized here. Therefore, lncRNAs were chosen for this purpose and their expression level and interaction with other signaling networks in prostate cancer progression were examined.

**Key scientific concepts of review:**

The aberrant expression of lncRNAs in prostate cancer has been well-documented and progression rate of tumor cells are regulated via affecting STAT3, NF-κB, Wnt, PI3K/Akt and PTEN, among other molecular pathways. Furthermore, lncRNAs regulate radio-resistance and chemo-resistance features of prostate tumor cells. Overexpression of tumor-promoting lncRNAs such as HOXD-AS1 and CCAT1 can result in drug resistance. Besides, lncRNAs can induce immune evasion of prostate cancer via upregulating PD-1. Pharmacological compounds such as quercetin and curcumin have been applied for targeting lncRNAs. Furthermore, siRNA tool can reduce expression of lncRNAs thereby suppressing prostate cancer progression. Prognosis and diagnosis of prostate tumor at clinical course can be evaluated by lncRNAs. The expression level of exosomal lncRNAs such as lncRNA-p21 can be investigated in serum of prostate cancer patients as a reliable biomarker.

## Background

Prostate is a walnut-sized reproductive organ located within the pelvic canal caudal to the urinary bladder and cranial to penis. The incidence of prostate cancer is high among men with 1 in 7 men in US and 1 in 25 worldwide diagnosed with this malignant condition in their lifetime [1, 2]. The enlargement of prostate that occurs with aging is called benign prostatic hyperplasia (BPH) and is associated with symptoms including polyuria observed in men over 60 years of age [3]. Due to similarities in histopathological and molecular presentations, BPH is considered as a phase in prostate tumor initiation. However, exact underlying mechanisms responsible for prostate tumor development from BPH have not been well understood [4, 5]. The incidence rate of prostate cancer is higher in developed countries due to availability of prostate specific antigen (PSA) testing for its diagnosis [6, 7]. Prostate tumor is among malignant tumors in men and newly published statistics demonstrate that it has an increase in incidence rate compared to 2020 with 248,530 people diagnosed resulting to 34,130 deaths [8]. Thanks to advancement in the field of medicine in recent years, particularly in developed countries, a significant improvement in survival and prognosis of prostate tumor patients has been observed. This can be observed in the 5-year survival rate of prostate tumor patients which stood at 97.8% in 2016, a significantly better record compared to 66.9% in 1975 [1]. Age, race, genetics, family history, obesity, and smoking, among the most common ones are risk factors of prostate tumor development [9–11]. If the treatment of prostate cancer fails, it progresses to a new form known as castration-resistant prostate cancer (CRPC) that is a problematic issue in clinical course and some major genes including androgen receptor (*AR*), *TP53*, *RB1*, *PTEN* and DNA damage repair (*DDR*) undergo mutations in this form of prostate cancer [12–14].

There are a variety of modalities in prostate tumor therapy. Surgery is beneficial in initial steps of prostate cancer. For advanced and metastatic forms of prostate cancer, chemotherapy and its combination with radiotherapy are utilized. Furthermore, due to dependence of prostate cancer cells on androgens, androgen-deprivation therapy (ADT) is extensively applied in its treatment. Immunotherapy including using immune checkpoint inhibitors, antibody-mediated radioimmunotherapy, antibody drug conjugates and bispecific antibodies is a new promising option in prostate cancer therapy [15–21]. However, due to the aggressive nature of prostate cancer cells, they acquire resistance to different therapies [22, 23]. They can activate tumor-promoting signaling pathways to induce chemoresistance, radio-resistance, ADT resistance and immune-resistance [24–30]. Therefore, strategies should be applied in reversing therapy resistance in prostate tumor, and this goal is achieved using pharmacological and genetic interventions [31–35]. Due to advances in field of genetics and bioinformatics, such molecular pathways have been recognized. Wnt, STAT3, Hedgehog (Hh), phosphatase and tensin homolog (PTEN), PI3K/Akt and NF-κB and SPOP are among the signaling networks undergoing abnormal expression in prostate cancer [36–44]. Noteworthy, non-coding RNAs (ncRNAs) are in special attention in prostate cancer due to their dual role in increasing/suppressing tumor progression [45–50].

Here, function of lncRNAs in prostate tumor is described in detail. It is started by an introduction about long non-coding RNAs (lncRNAs), their biogenesis and biological as well as their pathological functions. Then, we specifically discuss role of lncRNAs in progression rate (growth and migration), chemoresistance and radio-resistance of prostate tumor cells. Furthermore, role of lncRNAs as upstream mediators in regulation of major molecular pathways in prostate cancer is discussed. Finally, we describe currently applied therapeutics in targeting lncRNAs for prostate cancer therapy.

### LncRNAs: Biogenesis and role in oncology

**Fig. 1:**
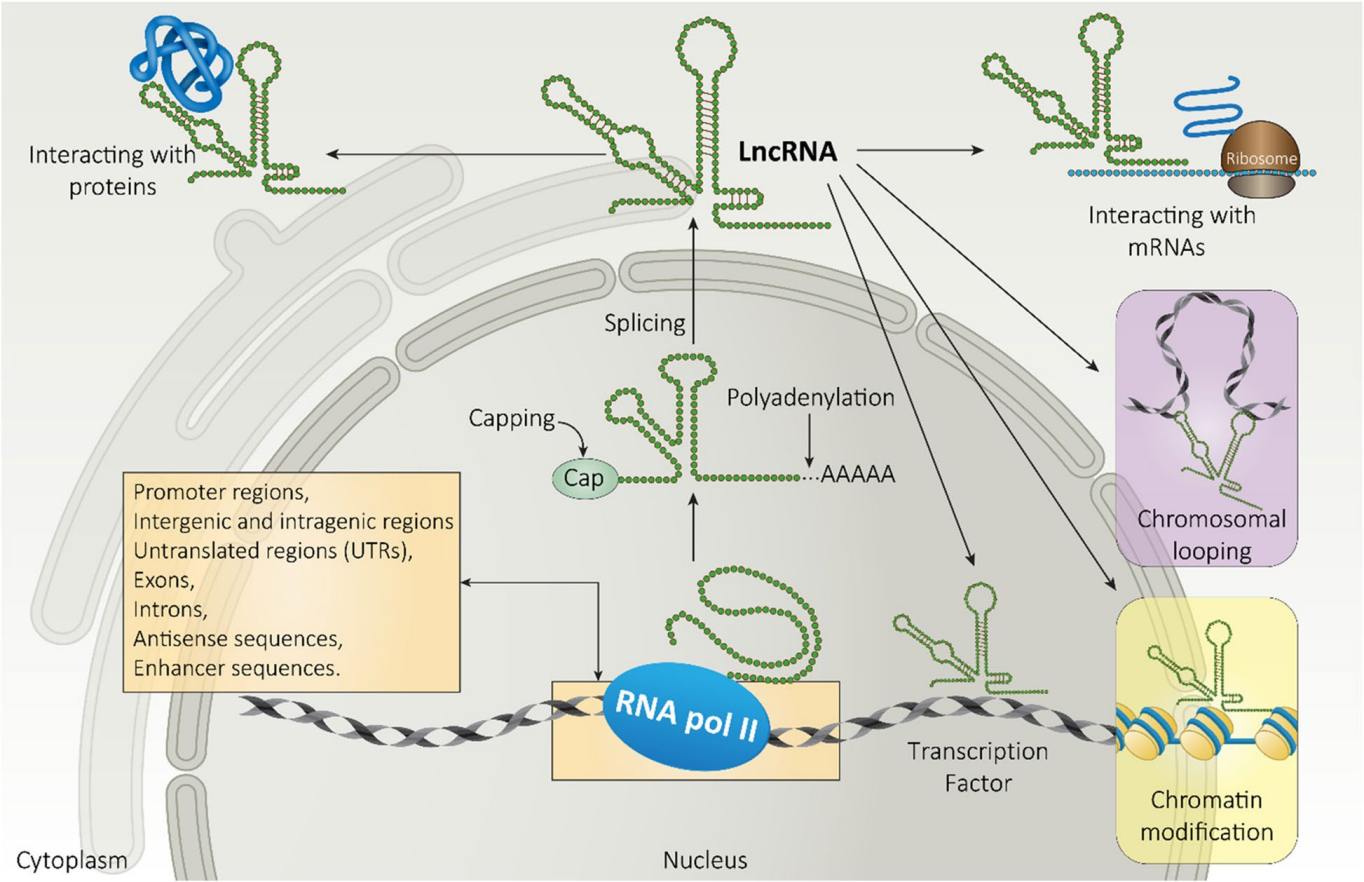
An overview of lncRNA function in affecting downstream targets. RNA polymerase II is involved in generation of lncRNAs and they participate in various functions in cells such as miRNA sponge, protein interaction and chromatin modification

It has been reported that less than 2% of human genome is made up of genes encoding proteins, and other 98% of genome is transcribed to RNA without following the way to encoding proteins [51–55]. Although ncRNAs were considered as junk parts of genome, now it is obvious that ncRNAs possess functional roles in cells [56–62]. ncRNAs lack lengthy open reading frames and are divided according to their size. Small ncRNAs are non-coding transcripts with length less than 200 nucleotides and include miRNAs, siRNA and piRNA. On the other hand, RNA molecules with length more than 200 nucleotides are known as lncRNAs. Currently, up to 100,000 lncRNAs have been identified [63]. LncRNAs are uniquely expressed in various tissues and specific cancer types [64]. The inability of lncRNAs to encode proteins is due to lack of open reading frame (ORF) [65]. Mutations in ncRNAs are responsible for development of human cancer [66]. It appears that lncRNAs can be transcribed by RNA polymerase II, capped, polyadenylated and spliced [67]. The biogenesis of lncRNAs can be performed from promoter regions, exons, antisense sequences, enhancer sequences, untranslated regions (UTRs) such as 3^/^ and 5^/^, introns, intergenic and intragenic regions of genome. Furthermore, lncRNAs can affect expression of their target using different actions. LncRNAs are able to function as signal, decoy, guide, scaffold and miRNA modulator in affecting biological processes and preserving homeostasis [68]. Figure 1 provides a schematic representation of lncRNA function in cells.

The function of lncRNAs is dependent on their location in cytoplasm or nucleus of cells. Increasing evidence demonstrates that lncRNAs located in nucleus are involved in gene modulation at epigenetic and transcription levels including histone modification, DNA methylation, chromatin remodeling, and interacting with proteins and transcription factors in nucleus [69–79]. On the other hand, there are lncRNAs located in cytoplasm that transcriptionally and post-transcriptionally modulate gene expression. These kinds of lncRNAs can interact with miRNAs (acting as competitive endogenous RNA (ceRNA)), affecting proteins in cytoplasm and modulating RNA metabolism [80–84]. Due to these vital functional roles of lncRNAs in cells, lncRNAs regulate growth, invasion, and drug resistance of tumor [85–91]. Recent studies reveal that lncRNAs are master regulators of signaling networks in cancer [92–95]. The lncRNAs usually affect miRNAs in tumors, and by affecting miRNA expression, lncRNAs affect survival and migration of cancer cells [96–98]. Furthermore, lncRNAs with tumor-promoting role such as CCAT2 can prevent apoptosis in cancer cells [99]. Importantly, lncRNAs can promote infiltration of immune cells such as B cells, T cells (both CD8+ and CD4+ T cells), neutrophils and dendritic cells in promoting anti-tumor immunity against cancer cells [100].

### LncRNAs in regulation of major molecular pathways

#### MicroRNAs

miRNAs are considered as short endogenous ncRNAs that can enhance or decrease expression of target messenger RNA (mRNA) by binding to 5^/^-UTR and 3^/^-UTR, respectively [101–103]. A miRNA can affect expression of different genes [104, 105]. Noteworthy, there are upstream mediators of miRNAs including lncRNAs that can reduce miRNA expression via sponging [106, 107]. Increasing evidence reveals dysregulation of miRNA expression in prostate cancer and association with malignant behavior of tumor cells [108–112]. In this section, we examine lncRNA impact on miRNAs in prostate tumor and its association with malignant behavior of cancer cells. Importantly, most of the works have focused on tumor-promoting lncRNAs. However, there are some studies evaluating role of tumor-suppressor lncRNAs in regulating miRNA expression in prostate cancer.

#### Tumor-promoting lncRNAs

LncRNA CCAT1 is considered as tumor-promoting factor that its role in various cancers have been discussed. CCAT1 increases endometrial cancer proliferation, while it down-regulates expression level of estrogen receptor-alpha (ERα) and its related molecular networks [113]. Increasing evidence demonstrates regulatory impact of lncRNA CCAT1 on miRNA expression in different cancers, so that CCAT1 can regulate miRNA-181a-5p and miRNA-138-5p in colorectal and pancreatic cancers, respectively for affecting progression [114, 115]. CCAT1 promotes tumor proliferation and progression in prostate tumor. For this purpose, CCAT1 interacts with miRNA-28-5p in cytoplasm (reduction in expression level) and paves the way for prostate cancer progression [116]. Noteworthy, lncRNAs can be affected by other upstream mediators in prostate cancer to mediate their regulatory impact on miRNAs. Such phenomenon occurs for lncRNA FOXP4-AS1 that prevents apoptosis in prostate tumor cells and significantly increases growth and metastasis. Paired box 5 (PAX5) is capable of triggering FOXP4-AS1 expression that in turn, functions as ceRNA for miRNA-3184-5p, leading to post-transcriptional regulation of FOXP4 and increasing its expression in favor of prostate cancer progression [117]. The regulation of lncRNAs by upstream mediators and its association with miRNA expression led to emergence of complicated molecular pathways, requiring more examination in further experiments.

LncRNA LINC00665 is a new emerging factor in cancer with crucial role in regulating various molecular pathways. Although there is evidence demonstrating that LINC00665 inhibits glioma progression via STAU1-mediated mRNA degradation [118], another experiment highlights that fact that LINC00665 overexpression is responsible for reduced overall survival of prostate cancer patients [119]. Therefore, LINC00665 possesses a tumor-promoting role of prostate cancer and can be considered as a prognostic and diagnostic tool. The overexpression of staphylococcal nuclease and Tudor domain containing 1 (SND1) is in favor of prostate cancer progression, and miRNA-1224-5p down-regulates SND1 expression in triggering cancer elimination. It has been reported that LINC00665 enhances tumor propagation, proliferation and metastasis via sponging miRNA-1224-5p and subsequent upregulation of SND1 [120]. Therefore, miRNAs are well-known downstream targets of lncRNAs, and tumor-promoting lncRNAs can affect their expression via sponging in mediating prostate cancer progression [117, 121].

LncRNA SNHG4 is an oncogenic factor in different cancers. LncRNA SNHG4 has multi-targeting ability and affects various mechanisms in promoting tumor malignancy. SNHG4 overexpression in gastric cancer leads to RRM2 upregulation via miRNA-204-5p down-regulation to prevent cell cycle arrest and to enhance growth and metastasis of tumor cells [122]. LncRNA SNHG4 is involved in increasing metastasis of gastric tumor cells via EMT induction by sponging miRNA-204-5p [123] and it also mediates immune evasion of cancer cells [124]. A same phenomenon occurs in prostate cancer and SNHG4 undergoes upregulation by an upstream mediator known as SP1. Then, SNHG4 promotes ZIC5 expression via miRNA-377 sponging to enhance survival of tumor cells and increase malignant behavior [125]. In case of recognizing a tumor-promoting lncRNA, the best strategy is its knock-down to diminish prostate cancer progression. For instance, silencing lncRNA TUG1 is beneficial in prostate cancer suppression and inducing radio-sensitivity via miRNA-139-5p overexpression and subsequent overexpression of SMC1A [126].

The capability of prostate tumor cells in mediating chemoresistance should be overcome [127]. LncRNA and miRNA interaction determines drug resistance in prostate tumor. The overexpression of lncRNA NEAT1 induces docetaxel resistance in prostate tumor. miRNA-34a-5p and miRNA-204-5p undergo down-regulation in prostate cancer and increasing their expression elevates chemosensitivity via preventing ACSL4 expression. As an upstream mediator, lncRNA NEAT1 down-regulates expression level of both miRNA-34a-5p and miRNA-204-5p to elevate ACSL4 expressions, leading to docetaxel resistance of prostate tumor cells [128].

#### Tumor-suppressor lncRNAs

LncRNA H19 is encoded by *H19* gene located on chromosome 11q15.5 [129]. Except skeletal muscle, H19 demonstrates a decrease in expression in most of the tissues [130, 131]. H19 overexpression is in favor of tumor progression by enhancing metastasis, triggering EMT and regulating molecular pathways such as miRNAs [121, 132, 133]. However, H19 is an anti-tumor factor in prostate cancer. There is a positive relationship between H19 and miRNA-675 in prostate cancer. By promoting miRNA-675 expression, H19 reduces TGF-β levels, leading to metastasis suppression of prostate cancer cells [134]. LncRNA MEG3 is another factor that its role in regulating miRNA expression in prostate cancer has been investigated. MEG3 has a similar role in other cancers such as ovarian cancer that can suppress progression and promote drug sensitivity [135, 136]. In prostate tumor cells and tissues, MEG3 expression undergoes down-regulation. Increasing MEG3 expression is associated with miRNA-9-5p down-regulation and subsequent increase in expression level of QKI-5, as downstream of miRNA-9-5p. This axis significantly suppresses growth and invasion of prostate tumor cells and induces apoptotic cell death [137].

ZEB1 mediates malignant behavior of prostate cancer cells. ZEB1 down-regulation is associated with a reduction in stemness of prostate tumor [138]. Furthermore, overexpression of ZEB1 promotes growth and metastasis as well as induces drug resistance in prostate cancer [139]. LncRNA IUR appears to suppress metastasis of prostate cancer cells. For this purpose, lncRNA IUR decreases ZEB1 expression via miRNA-200 upregulation to impair prostate cancer progression [140]. Restoring expression level of tumor-suppressor lncRNAs stimulates apoptosis and interferes with proliferation of prostate cancer cells [141].

**Fig. 2:**
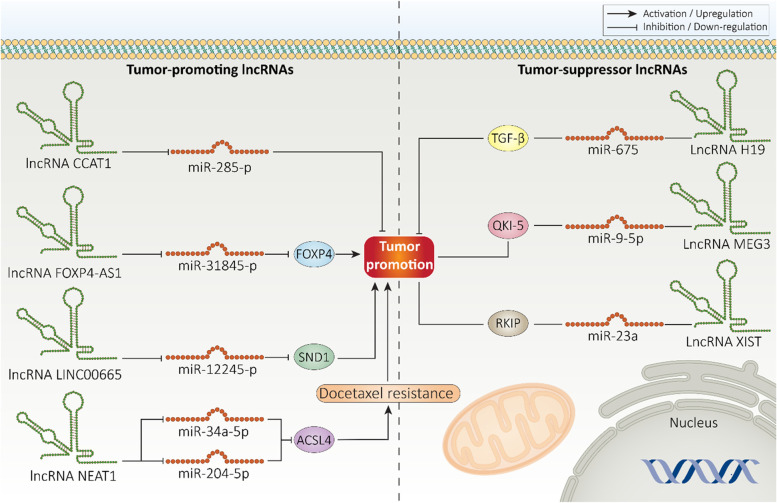
The lncRNAs regulating miRNAs in prostate cancer. LncRNAs reduce the expression level of target miRNAs via sponging. The tumor progression including proliferation and invasion, as well as drug resistance are modulated by lncRNA/miRNA axis in prostate cancer. Regulating expression level of lncRNAs or miRNAs is beneficial in impairing progression of prostate cancer cells

As more experiments are performed, more lncRNAs involved in prostate cancer progression/inhibition are identified. The interesting point is that lncRNA role is context-dependent and a certain lncRNA may possess various functions in different cancer types [142–144]. Hence, the exact role of each lncRNA in different cancers should be explored. LncRNA XIST is such factor that demonstrates tumor-promoting role in gastric and ovarian cancers via regulating miRNA expression [145, 146], while it has tumor-suppressor role in prostate cancer. Enhancing XIST expression diminishes miRNA-23a expression via sponging to upregulate RKIP expression at post-transcriptional level, resulting in reduced prostate cancer growth and migration [147]. These experiments clearly highlight role of lncRNAs in regulating miRNA expression and affecting prostate cancer progression [148]. However, we are still a long way from understanding the full potential of lncRNAs in prostate cancer progression/inhibition (Table 1 and Figure 2).

**Table 1:** LncRNAs regulating miRNAs in prostate cancer

LncRNA	Signaling network	Major impacts	Refs
TUC338	MiRNA-466	Acting as tumor-promoting factor TUC338 down-regulates miRNA-466 expression to increase progression of prostate cancer	[149]
IUR	MiRNA-200/ZEB1	Increased expression of miRNA-200 by lncRNA IUR Subsequent inhibition of ZEB1 in inhibiting cancer invasion	[140]
BRE-AS1	MiRNA-145-5p	Acting as tumor-suppressor factor BRE-AS1 enhances miRNA-145-5p expression to stimulate apoptosis in prostate cancer cells	[141]
TUG1	MiRNA-139-5p/SMC1A	Reduced miRNA-139-5p expression by lncRNA TUG1 SMC1A upregulation Triggering radio-resistance feature of prostate cancer	[126]
HOXA-AS2	MiRNA-509-3p/PBX3	Enhancing progression of prostate cancer Reducing miRNA-509-3p expression via sponging to enhance PBX3 expression	[150]
PVT1	MiRNA-146a	Reducing expression level of miRNA-146a by triggering methylation of CpG islands Enhancing prostate cancer cell viability Apoptosis inhibition	[151]
UCA1	MiRNA-331-3p/EIF4G1	Increased expression of UCA1 and EIF4G1 in prostate cancer Reduced expression of miRNA-331-3p by UCA1 via sponging Mediating radio-resistance	[152]
SNHG1	MiRNA-199a-3p/CDK7	Increasing growth and survival of prostate cancer cells Triggering cell cycle progression Reducing miRNA-199a-3p expression to upregulate CDK7	[153]
SNHG4	MiRNA-377/ZIC5	Overexpression of SNHG4 in prostate cancer by SP1 Reducing miRNA-377 expression by acting as ceRNA Increasing ZIC5 expression to mediate proliferation and invasion	[125]
OGFRP1	MiRNA-124-3p/SARM1	Decreasing miRNA-124-3p expression by acting as ceRNA SARM1 upregulation Mediating malignant behavior of prostate tumor cells	[154]
KCNQ1OT1	MiRNA-211-5p/CHI3L1	Overexpression of lncRNA in prostate cancer cells and tissues Decreasing miRNA-211-5p levels to increase CHI3L1 levels Increasing growth and migration	[155]
MALAT1	MiRNA-320b/AR	Reduction in miRNA-320b expression by MALAT1 to induce AR signaling Increasing cell cycle progression	[156]
FAM83H-AS1	MiRNA-15a/CCNE2	Sponging miRNA-15a to increase CCNE2 expression Promoting growth and cell cycle progression of prostate tumor	[157]
ANRIL	Let-7a/TGF-β1/Smad	Reducing expression level of Let-7a to induce TGF-β signaling Increasing metastasis and invasion	[158]
TTTY15	MiRNA-29a-3p/DVL3	Positive association with tumor progression Increasing DVL3 expression via miRNA-29a-3p down-regulation	[159]
BLACAT1	MiRNA-29a-3p/DVL3	The miRNA-29a-3p expression inhibition by BLACAT1 and subsequent increase in DVL3 levels Mediating prostate tumor progression	[160]

#### Wnt signaling

Another promising target in cancer suppression is Wnt/β-catenin [161–163]. Briefly, Wnt signaling activation occurs by attachment of Wnt ligand to cell membrane receptors, known as Frizzled (Fz). Besides, Wnt ligands can bind to LRP families on cell membrane to induce Wnt signaling. Upon activation, β-catenin translocates into nucleus to stimulate downstream targets involved in cancer progression. However, in normal conditions, GSK-3β participates in degrading β-catenin and translocation to nucleus is inhibited [164, 165]. Activation of Wnt signaling can mediate growth, metastasis and therapy resistance of prostate tumor [166–168]. LncRNAs have been shown to exert regulatory influence on Wnt signaling in prostate cancer. Wnt2B activation results in EMT induction in prostate cancer. miRNA-324-3p diminishes Wnt2B expression to inhibit EMT-mediated migration of prostate tumor. LncRNA SNHG7, owing to its tumor-promoting role, can reduce miRNA-324-3p expression to elevate Wnt2B expression, resulting in EMT and progression of prostate cancer cells. Silencing SNHG7 significantly impairs progression of prostate tumor, highlighting role of this lncRNA in metastasis via Wnt signaling activation [169].

LncRNA noncoding RNA activated by DNA damage (NORAD) is another factor capable of regulating Wnt signaling and prostate cancer progression. Overall, NORAD is involved in development of different cancers such as lung cancer, ovarian cancer and osteosarcoma [170–172]. It appears that NORAD is a critical regulator of miRNAs in different cancers [173]. In order to affect Wnt signaling in prostate cancer, NORAD targets miRNA-30a-5p. By binding to miRNA-30a-5p and acting as a ceRNA, NORAD upregulates expression level of RAB11A as a member of RAS oncogene family, resulting in Wnt/β-catenin activation and subsequent increase in metastasis of prostate cancer cells via EMT induction [174].

Androgen-independent prostate cancer (AIPC) is a complex condition in which prostate cancer cells do not depend on androgen for their progression and ADT is not effective [175]. It has been reported that genomic alterations and cellular events participate in development of AIPC [176, 177]. Recent study has shown that lncRNAs can regulate Wnt signaling to affect progression of AIPC cells. LncRNA LEF1-AS1 shows overexpression in APIC cells and tissues that subsequently promotes proliferation and invasion. In this way, lncRNA LEF1-AS1 increases expression level of FZD2 to activate Wnt signaling. Furthermore, LEF1-AS1 induces GSK-3β phosphorylation at Serine 9 to prevent β-catenin degradation [178].

The role of lncRNA/Wnt axis in therapy response and progression of prostate cancer cells has been examined. The sensitivity of prostate tumor to cisplatin diminishes upon Wnt stimulation. miRNA-425-5p upregulation can increase cisplatin-mediated apoptosis via β-catenin down-regulation [179]. LncRNA HOTTIP is capable of promoting proliferation of prostate tumor and triggering cisplatin resistance. Knock-down of lncRNA HOTTIP inhibits Wnt pathway, resulting in cell death, cell cycle arrest and cisplatin sensitivity of prostate cancer cells [180]. Therefore, lncRNAs are potent regulators of Wnt signaling in prostate cancer and identification of their interaction is of importance in understanding mechanisms involved in prostate cancer progression/inhibition. Furthermore, experiments have focused on tumor-promoting lncRNAs inducing Wnt signaling, and function of tumor-suppressor lncRNAs in Wnt modulation should be explored [181–185].

#### STAT3 signaling

STAT3 protein has 770 amino acids with 6 functionally conserved domains mediating its biological roles [186–188]. A variety of ligands have been identified for STAT3 signaling including Janus kinase (JAK), tyrosine kinases and cytokines that can result in STAT3 phosphorylation at tyrosine 705 and serine 727, leading to nuclear translocation, DNA binding and affecting downstream targets [189–191]. Upregulation of STAT3 promotes metastasis of prostate tumor to bone [192]. STAT3 signaling activation elevates CRPC cell viability and metastasis [193]. Exposing CRPC cells to enzalutamide (Enz) elevates lncRNA-p21 expression that is required for neuroendocrine differentiation (NED). Enz induces AR signaling to promote lncRNA-p21 expression that in turn, upregulates expression level of EZH2 which is required for suppressing STAT3 signaling by lncRNA-p21. In this way, lncRNA-p21 changes EZH2 function from histone-methyltransferase to non-histone methyltransferase to induce STAT3 methylation, leading to NED and CRPC suppression [194]. This study demonstrates that lncRNAs can indirectly affect STAT3 expression by targeting their upstream mediators. miRNAs are other upstream mediators of STAT3 in cancer [195, 196]. LINC00473 reduces expression level of miRNA-195-5p to enhance expression level of SEPT2 in prostate cancer. In turn, SEPT2 induces JAK/STAT3 signaling to dually increase growth and viability of prostate tumor [197].

#### PTEN/PI3K/Akt/mTOR signaling

PTEN is a tumor-suppressor located on chromosome 10 with mutation in various cancers [198–200]. Owing to its lipid-phosphatase activity, PTEN diminishes cellular levels of phosphatidylinositol-3,4,5-phosphate (PIP3) that is considered as a seconder messenger in different biological and molecular mechanisms [201]. By reducing PIP3 levels, PTEN inhibits PI3K signaling and its downstream axis Akt/mTOR that is responsible for cancer progression [196, 202]. Increasing evidence has confirmed role of PTEN signaling in prostate cancer. Polymorphisms in *PTEN* gene is responsible for extracapsular extension in prostate cancer [203]. In CRPC cells, the phosphorylation of PTEN by LIMK2 results in its degradation, paving the way for cancer progression [204]. Besides, activation of PI3K/Akt axis prevents ferroptosis in prostate tumor [205], and mediates therapy resistance [206]. LncRNAs are potent modulators of PTEN and PI3K/Akt in prostate tumor. Noteworthy, for promoting progression of prostate cancer, lncRNAs should be capable of decreasing PTEN expression. LncRNA MCM3AP-AS1 has overexpression in prostate tumor and its knockdown prevents tumor progression. Mechanistically, MCM3AP-AS1 down-regulates miRNA-543-3p to inhibit PTEN, resulting in Akt signaling activation and further promotion in progression of prostate cancer cells [207]. Decreasing expression level of tumor-promoting lncRNAs such as PlncRNA-1 enhances PTEN expression to suppress Akt signaling and prostate cancer progression [208]. By inducing PI3K/Akt/mTOR axis, lncRNA LINC01296 enhances proliferation and survival. This axis can be considered as a biomarker in prostate cancer, in which its activation provides poor prognosis in prostate cancer [209].

Similar to other molecular pathways discussed before, activation of PI3K/Akt signaling is responsible for drug resistance trait of prostate cancer [210]. Overexpression of lncRNA PCAT6 occurs in prostate cancer cells resistant to 5-flourouracil (5-FU). In this way, PCAT6 down-regulates miRNA-204 expression to induce HMGA2/PI3K axis, resulting in drug resistance [211]. As miRNAs play a remarkable role in PI3K/Akt regulation in cancer [212], their regulation by lncRNAs occurs in prostate cancer. It has been reported that lncRNA HCG11 overexpression significantly stimulates apoptosis and simultaneously, inhibits prostate tumor progression. HCG11 is capable of miRNA-543 down-regulation to inhibit PI3K/Akt signaling in impairing prostate cancer growth [213]. The impact of lncRNA/PI3K/Akt axis on prostate cancer progression is attributed to downstream targets of this signaling network. The expression level of lncRNA DANCR enhances in prostate cancer and induces EMT-mediated metastasis. By reducing expression level of miRNA-185-5p, DANCR increases LIM and SH3 protein 1 (LASP1), resulting in FAK/PI3K/Akt axis induction. Then, Akt phosphorylates GSK-3β to stimulate Snail expression in promoting prostate tumor progression [214]. Overall, modulation of PI3K/Akt signaling by lncRNAs occurs in prostate cancer [215], and therapeutic targeting of lncRNAs, using pharmacological or genetic interventions, can result in cancer inhibition.

#### Notch signaling

Notch signaling is a new emerging target in prostate cancer due to its tumor-promoting function. Notch1 can promote expression levels of MMP-2 and MMP-9 in increasing progression and metastasis of prostate cancer cells. As anti-cancer agent, rubimaillin suppresses Notch signaling to down-regulate MMP-2 and MMP-9 expressions in inhibiting growth and invasion of prostate cancer cells [216]. Aspartate β-hydroxylase is involved in castration-resistant prostate cancer via activation of Notch signaling [217]. Overexpression of Notch1 is linked to EMT stimulation in enhancing metastasis of prostate tumor cells [218]. Furthermore, Notch signaling stimulates drug resistance in prostate cancer and its inhibition is of importance in reversing chemoresistance [219]. Studies have demonstrated interaction between lncRNAs and Notch signaling in regulating prostate cancer progression. HIF-1α functions as upstream mediator to stimulate Notch1 signaling in prostate cancer. LncRNA GHET1 reduces KLF2 expression to trigger HIF-1α/Notch1 signaling in increasing prostate cancer progression. Notably, silencing GHET1 promotes KLF2 expression, leading to HIF-1α/Notch1 inhibition and subsequent decrease in prostate cancer progression [220]. Future studies will shed more light on the interaction between lncRNAs and Notch signaling in prostate cancer.

#### ***NF-***κ***B signaling***

NF-κB contains five subunits such as NF-κB1, NF-κB2, c-Rel, RelA and RelB [221, 222]. It has two main pathways including classical pathway for which RelA and cRel play critical role, and alternative pathway that applies to RelB containing dimers [223, 224]. Due to tumor-promoting role of NF-κB signaling in cancer, its synthetic and natural inhibitors have been developed [225, 226]. ncRNAs are considered as potent regulators of NF-κB signaling in cancer [227]. The increasing evidence demonstrates that NF-κB signaling activation can significantly promote progression of prostate cancer cells and induces their resistance to therapy [193, 228, 229]. In this section, we provide a discussion of lncRNAs role in NF-κB regulation in prostate cancer.

**Fig. 3:**
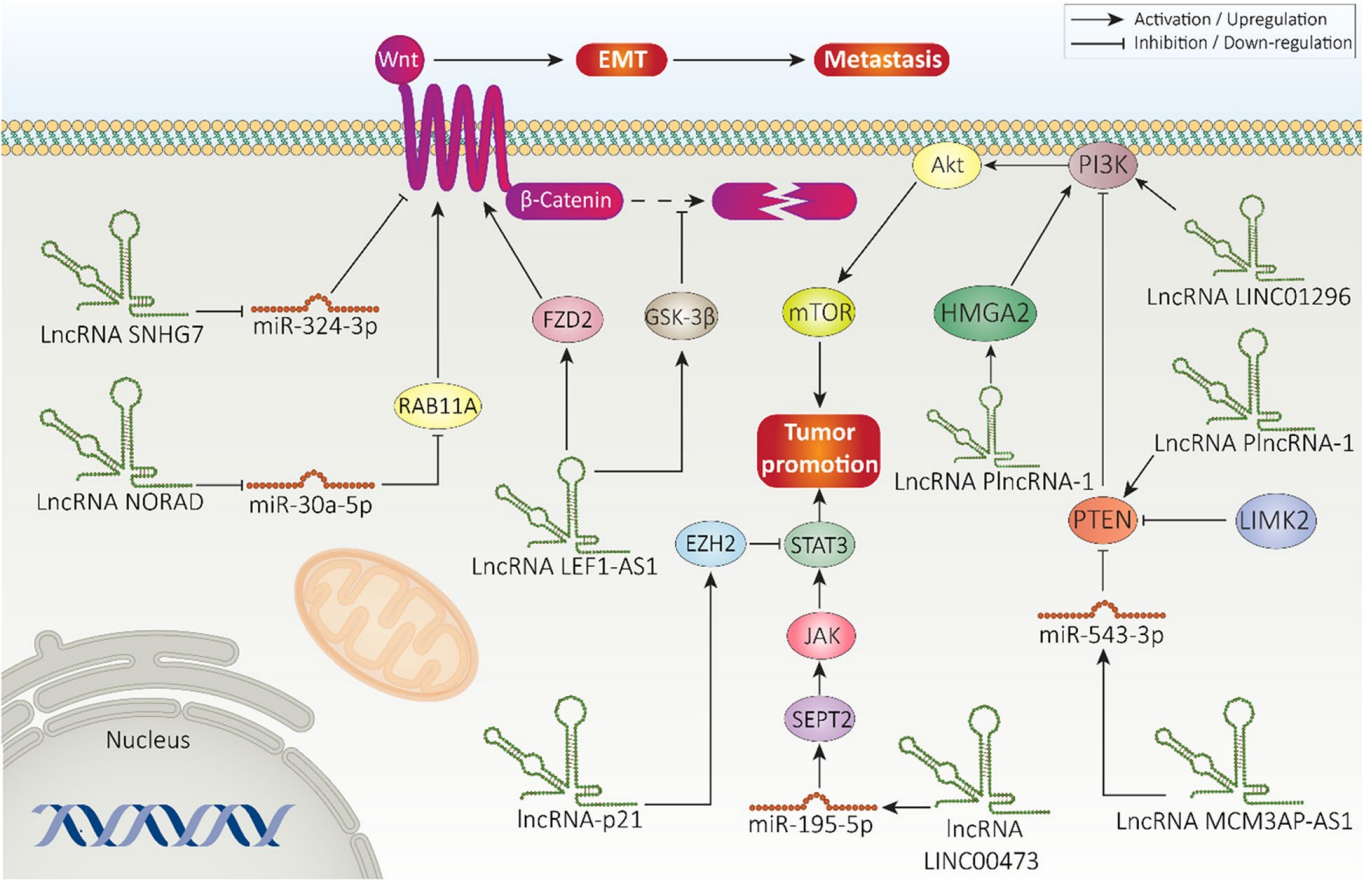
LncRNAs in regulation of other molecular pathways in prostate cancer. PTEN, STAT3, mTOR and EZH2 are main molecular pathways involved in regulating prostate cancer progression. LncRNAs can directly interact with aforementioned factors or target miRNAs in affecting their expression level

The activation of NF-κB signaling is mediated via cytokines such as tumor necrosis factor-a (TNF-α) and interleukin-1 (IL-1), among others [230, 231]. These factors stimulate IκB kinase complex (IKK), consisting of the catalytic IKKα and IKKβ subunits [232, 233]. IKK complex induces proteasomal degradation of IκBα protein via phosphorylation to release NF-κB, resulting in its nuclear translocation and activation of downstream targets [234–236]. As a tumor-suppressor factor, lncRNA DRAIC inhibits capacity of IKK complex in phosphorylating IκBα, resulting in NF-κB signaling inhibition and decreased progression of prostate cancer cells [237]. On the other hand, there are lncRNAs capable of inducing NF-κB signaling. It has been reported that lncRNA cardiac hypertrophy-related factor (CHRF) can upregulate miRNA-10b expression to induce NF-κB signaling and promote progression of prostate cancer cells. Silencing lncRNA CHRF significantly inhibits metastasis (EMT) and proliferation [238]. For activation of NF-κB signaling in prostate cancer, a complex containing different factors should be formed or disrupted. PH and leucine-rich repeat protein phosphatase (PHLPP) can interact with FKBP51 in regulating IKKα level. LncRNA PCAT1 induces NF-κB signaling to enhance CRPC progression via dissecting PHLPP from FKBP51/IKKα complex [239]. To date, a few experiments have explored role of lncRNAs in regulating NF-κB signaling in prostate cancer. However, these studies are in agreement with the fact that NF-κB and its components such as IKKα are regulated by lncRNAs and this axis affects both metastasis and growth of prostate cancer cells. Future studies can focus on the role of lncRNA/NF-κB axis in therapy response of prostate cancer. Figure 3 provides a summary of molecular pathways regulated by lncRNAs in prostate cancer therapy.

### LncRNAs and molecular mechanisms

#### Role in proliferation

Cancer cells demonstrate rapid proliferation that requires high amount of energy provided by glucose uptake and consumption [240]. One of the distinct differences between normal and cancer cells is their way of energy production, in that cancer cells depends on glucose metabolism instead of oxidative phosphorylation in mitochondria [241]. Therefore, suppressing glycolysis or Warburg effect is a promising strategy in cancer therapy [242]. Glucose transporter-1 (GLUT-1) mediates translocation of glucose across cell membrane and its upregulation is associated with enhanced cancer progression, particularly prostate cancer [101, 243]. The glucose metabolism is affected by lncRNAs in prostate cancer. LncRNA SNHG16 possesses a tumor-promoting role that its overexpression stimulates glucose uptake and metabolism, leading to increased prostate cancer proliferation. Knock-down of SNHG16 significantly reduces GLUT-1 expression and prevents prostate cancer proliferation [244].

LncRNAs can regulate apoptosis in prostate cancer. Toll-like receptor (TLR) is an apoptosis-related pathway that its induction occurs in tumor microenvironment [245]. The activation of TLR signaling pathway occurs in prostate cancer to promote its progression [246]. LncRNA PART1 is capable of inducing TLR signaling and its downstream targets including TLR3, TNFSF10 and CXCL13 in apoptosis inhibition in prostate cancer. Silencing PART1 is associated with a decrease in prostate cancer proliferation and apoptosis induction [247]. Both *in vitro* and *in vivo* experiments have shown that overexpression of tumor-promoting lncRNAs can enhance prostate cancer proliferation and prevents apoptosis. By reducing miRNA-15a-5p expression, lncRNA PVT1 promotes KIF23 expression to prevent apoptosis in prostate cancer. Knock-down of PVT1 is correlated with apoptosis induction [248]. Overall, experiments have evaluated role of lncRNAs in regulating prostate cancer proliferation via affecting molecular pathways [249–251] that the major ones discussed in previous sections.

#### Role in metastasis

A high number of prostate cancer-related mortality arises from metastasis that is due to dissemination of cancer cells to distant organs including lung, liver, bone, and lymph nodes [252]. Bone metastasis is the most common complication of prostate cancer which subsequently, is associated with osteoblastic and osteolytic lesions [253]. Therefore, it is vital to identify factors involved in prostate cancer metastasis for the management of this malignant condition. Furthermore, the molecular pathways related to prostate cancer metastasis can be considered as biomarkers for prostate cancer prognosis [254, 255]. One of the molecular pathways involved in regulating prostate cancer metastasis is *NDRG1* gene that its down-regulation results in increased migration [256]. As a tumor-suppressor factor, lncRNA LINC00844 undergoes down-regulation in metastatic prostate cancer cells and is associated with poor prognosis. Mechanistically, LINC00844 mediates AR binding to chromatin and its expression is vital for promoting *NDRG1* gene expression in suppressing prostate cancer migration and invasion [257]. Increasing evidence has revealed role of transforming growth factor-beta (TGF-β) in mediating bone metastasis of prostate cancer cells via EMT induction [258, 259]. LncRNA prostate cancer-associated transcript 7 (PCAT7) is also called PCAN-R2 and located on chromosome 9q22.32. LncRNA PCAT7 is suggested to be involved in cancer progression [260, 261]. In prostate cancer, upregulation of PCAT7 enhances bone metastasis and aggressive behavior of prostate cancer cells via EMT induction. In this way, PCAT7 reduces miRNA-324-5p expression via sponging to enhance TGFBR1 expression, resulting in TGF-β/Smad axis stimulation. Furthermore, TGF-β signaling can form a positive feedback loop with PCAT7 to enhance its expression, resulting in EMT induction and bone metastasis of prostate cancer cells [262].

**Fig. 4:**
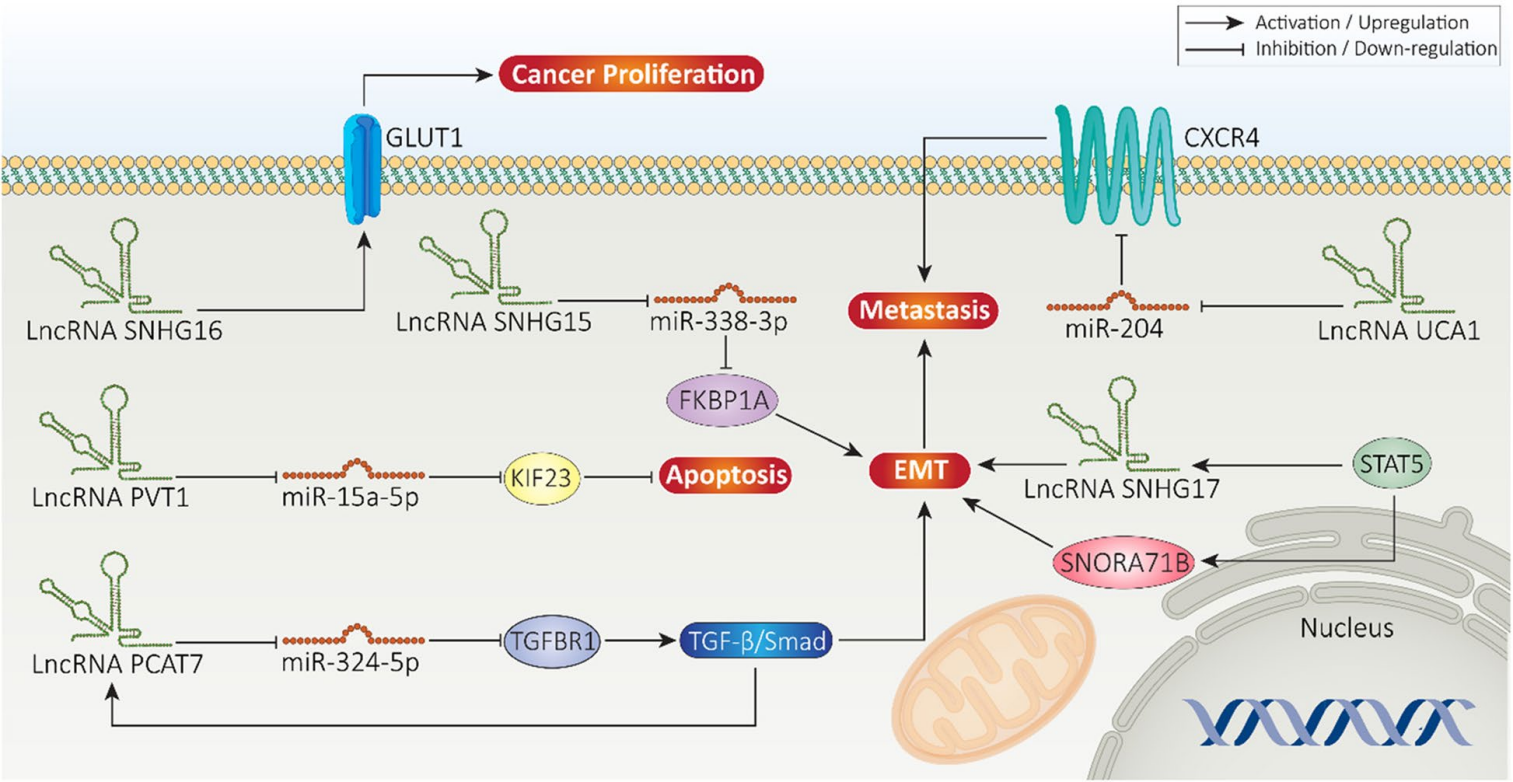
Role of lncRNAs in proliferation and metastasis of prostate cancer cells. EMT is responsible for increasing migration and invasion of prostate cancer cells. LncRNA SNH17 and PCAT7 are among the lncRNAs inducing EMT in increasing prostate cancer metastasis. Apoptosis induction and transfer of glucose into prostate cancer cells (GLUT1) are also modulated by lncRNAs

Another factor responsible for bone metastasis of prostate cancer is C-X-C chemokine receptor type 4 (CXCR-4) [263, 264]. The overexpression of CXCR4 occurs in different cancers and mediates their aggressive behavior [265–268]. In prostate cancer, CXCR4 upregulation is associated with poor prognosis and induces lymph node and bone metastasis [269]. LncRNA UCA1 can regulate CXCR4 expression in prostate cancer cells to affect their progression. By sponging miRNA-204, lncRNA UCA1 promotes expression level of CXCR4 to enhance metastasis of prostate cancer cells [270]. As it was mentioned, EMT induction is responsible for increased prostate cancer migration and invasion. EMT includes both morphological and cellular alterations [271]. At morphological level, epithelial cells that have low mobility, are transformed to mesenchymal cells with high migratory ability. At cellular level, a decrease occurs in E-cadherin level, while levels of N-cadherin and vimentin increase [55, 272]. In prostate cancer, STAT5A activates both lncRNA SNHG17 and SNORA71B to induce EMT and promote metastasis [273]. The same function is mediated by SNHG15 in prostate cancer that its overexpression significantly increases prostate cancer metastasis via EMT induction. Mechanistically, SNHG15 down-regulates miRNA-338-3p by acting as ceRNA to upregulate KBP prolyl isomerase 1A (FKBP1A), leading to EMT-mediated metastasis of prostate cancer [274]. Overall, lncRNAs are critical modulators of prostate cancer metastasis and more studies are needed to highlight other lncRNAs involved in promoting migration and invasion [275, 276]. Figure 4 highlights role of lncRNAs in regulating proliferation and migration of prostate cancer cells.

#### Role in therapy response

Although ADT is applied in prostate cancer therapy, it seems that these malignant cells can promote their progression via androgen-independent manner. Other kinds of therapies such as chemotherapy regimen with docetaxel and cabazitaxel and antiandrogens such as abiraterone and Enz are utilized in prostate cancer therapy [277–282]. However, it has been shown that prostate cancer cells can trigger chemoresistance [283, 284]. In respect to role of lncRNAs in regulating various molecular pathways in prostate cancer, these ncRNAs can affect drug resistance feature. Furthermore, prostate cancer cells can obtain resistance to radiotherapy [285]. The aim of this section is to examine role of lncRNAs in regulating therapy response of prostate cancer cells.

HOXD-AS1 is encoded by HOXD cluster gene and a recent experiment has evaluated its role in cancers. Overexpression of HOXD-AS1 enhances cyclin D1 expression via miRNA-526b-3p down-regulation, resulting in proliferation and metastasis of colorectal cancer cells [286]. By acting as ceRNA, lncRNA HOXD-AS1 promotes expression level of fibroblast growth factor 2 (FGF2) in mediating cervical cancer progression [287]. On the other hand, WD repeat domain 5 (WDR5) interacts with lncRNAs in maintaining chromatin activation [288]. In CRPC, silencing HOXD-AS1 impairs proliferation and increases sensitivity to chemotherapy. HOXD-AS1 recruits WDR5 to trigger histone H3 lysine 4 tri-methylation of target genes such as PLK1, AURKA, CDC25C, FOXM1 and UBE2C, leading to chemoresistance induction in prostate cancer [289]. Doxorubicin (DOX) is a well-known chemotherapeutic agent applied in cancer therapy. DOX administration stimulates apoptosis and cell cycle arrest via inhibiting topoisomerase activity [272, 290]. Prostate cancer cells have demonstrated DOX resistance by affecting various molecular pathways. p53 down-regulation and retinoic acid-related orphan nuclear receptor γ (RORγ) upregulation are among the factors involved in DOX resistance in prostate cancer [291, 292]. LncRNA LOXL1-AS1 is capable of promoting epidermal growth factor receptor (EGFR) in prostate cancer via miRNA-3et-7a-5p down-regulation to mediate DOX resistance. Silencing LOXL1-AS1 impairs proliferation and sensitizes prostate cancer cells to DOX-mediated apoptosis [293].

Paclitaxel (PTX) is another chemotherapy regimen used in cancer therapy including that of prostate. In respect to PTX resistance of prostate cancer cells, polymeric nanoparticles have been applied for targeted delivery of PTX [294]. Furthermore, activation of molecular mechanisms such as EMT stimulates PTX resistance [295]. LncRNA CCAT1 undergoes overexpression in PTX resistant-prostate cancer cells and prevents apoptosis. In this way, CCAT1 reduces miRNA-24-3p expression to upregulate fascin1 (FSCN1) expression, leading to prostate cancer proliferation, survival and PTX resistance [296]. Overall, drug resistance is a common feature of prostate cancer cells that is attributed to their aggressive behavior. Identification of lncRNAs and their downstream targets can pave the way to effective prostate cancer chemotherapy [297].

Radio-resistance is another problematic issue in prostate cancer therapy [298]. One of the molecular mechanisms involved in radio-resistance is autophagy. Briefly, autophagy is responsible for providing energy during starvation via degradation of amino acids and macromolecules. Furthermore, autophagy degrades aged organelles in cells. AMP-activated protein kinase (AMPK) and Beclin-1 are considered as inducers of autophagy, while mTOR signaling suppresses autophagy [299]. Recently, attention has been directed towards role of autophagy in cancer progression. Autophagy plays like a double-edged sword in cancer and can increase cancer malignancy [103]. Recently published experiments demonstrated that autophagy activation by upstream mediators such as Wnt, miRNA-129-5p and AMPK can result in radio-resistance [300–302]. On the other hand, there are studies showing that autophagy activation promotes radio-sensitivity [303, 304]. Therefore, more experiments are required to reveal exact role of autophagy in caner. LncRNA highly upregulated in liver cancer (HULC) has shown a tumor-promoting role in prostate cancer. The overexpression of HULC induces radio-resistance in prostate cancer and its silencing is correlated with cell cycle arrest at G0/G1 phase. HULC can inhibit autophagy via Beclin-1 down-regulation and triggering mTOR signaling. The autophagy inhibition by HULC sensitizes prostate cancer cells to irradiation by apoptosis induction through enhancing caspase-3 and Bax levels [305].

#### Role in immune regulation

Cancer cells are able to regulate various intrinsic and extrinsic biological pathways to ensure their adaptation to host defense. These adaptations include stimulation of tumor-promoting mechanisms, preventing cell death, angiogenesis induction, promoting migration and finally, triggering immune evasion [306]. Generally, natural killer (NK) and cytotoxic T cells (CTLs) are involved in anti-tumor immunity via apoptosis induction and mediating cell lysis [307]. However, cancer cells have obtained resistance to immune surveillance, and they are no longer responsive to immune system-mediated lysis. They can form an immunosuppressive microenvironment to escape anti-tumor immunity [308]. Immune evasion commonly occurs in prostate cancer, threatening efficacy of immunotherapy. In CRPC, Dickkopf-1 (DKK1) induces Wnt signaling, resulting in immune evasion [309]. It is worth mentioning that EMT induction and increased N-cadherin levels can reduce levels of cytotoxic T cells (CD8+), while they promote level of immunosuppressive regulatory T cells (CD4^+^/FOXP3^+^), triggering immune evasion of prostate cancer [310]. In this section, the regulatory impact of lncRNAs on immune system in prostate cancer is discussed.

One of the most well-known molecular pathways involved in immune evasion is programmed death-1 (PD-1) and its ligand, PD-L1. The tumor-suppressor factors are capable of regulating PD-L1 expression in prostate cancer. Retinoblastoma protein RB decreases expression level of PD-L1 to promote anti-tumor immunity and potential of radiotherapy in prostate cancer treatment [311]. The cyclin D-CDK4 can induce proteasomal degradation of PD-L1 in preventing immune evasion of prostate cancer [312]. Noteworthy, lncRNAs are considered as potent modulators of PD-L1 in cancer [313]. A recent experiment has shown that lncRNA KCNQ1QT1 induces escape of prostate cancer cells from immune surveillance. Normally, miRNA-15a binds to 3^/^-UTR of PD-L1 to reduce its expression, preventing apoptosis in CD8+ T cells and increasing their proliferation. Furthermore, miRNA-15a/PD-L1 axis enhances apoptosis induction in prostate cancer cells and impairs their proliferation and migration. It has been reported that lncRNA KCNQ1QT1 down-regulates miRNA-15a expression via sponging to induce PD-L1 signaling, increasing immune evasion of prostate cancer [314].

**Fig. 5:**
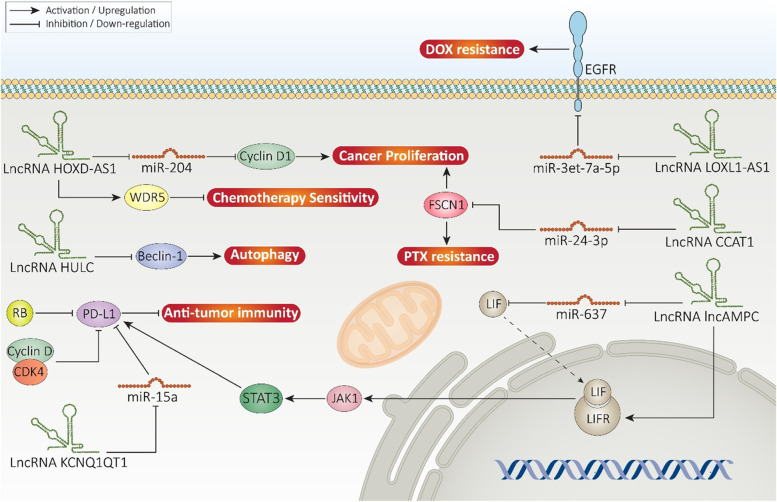
The lncRNAs regulate therapy response and immune system interactions in prostate cancer. The overexpression of tumor-promoting lncRNAs lead to drug resistance. Furthermore, overexpression of PD-L1 by lncRNAs can lead to immune escape in prostate cancer

The signaling networks involved in regulating PD-L1 expression in prostate cancer is of importance for developing novel therapeutics in near future. LIF is a pleiotropic cytokine with physiological functions in embryonic development [315]. Increasing evidence demonstrates tumor-promoting role of LIF in cancer and its potential in mediating therapy resistance and increasing self-renewal capacity of cancer-initiating cells [316, 317]. LIF can function as upstream mediator of JAK1/STAT3 signaling in preventing differentiation of cancer cells [318]. A recent experiment has shown how lncRNAs can regulate LIF/STAT3 axis in affecting immune response of prostate cancer cells. Upregulation of lncRNA lncAMPC enhances metastasis and immune evasion. The process is started from cytoplasm, where lncAMPC reduces expression level of miRNA-637 via sponging to enhance LIF expression. lncAMPC then translocates into nucleus to promote LIFR expression via decoying histone H1.2. The activation of LIF/LIFR axis stimulates JAK1/STAT3 signaling to preserve PD-L1 expression, leading to immune evasion of prostate cancer [319]. PD-1 inhibitors are of interest in cancer immunotherapy. However, upregulation of LIF can prevent infiltration of CD8+ T cells, impairing efficacy of anti-PD-1 therapy [320]. It appears that lncRNAs can affect infiltration of immune cells. LncRNA SNHG9 is considered as a tumor-promoting factor in prostate cancer that diminishes infiltration of T central memory (Tcm) cells and T helper cells, while it promotes infiltration of plasmacytoid dendritic cells (pDCs) and NK CD56 bright cells. Furthermore, overexpression of SNHG9 mediates poor prognosis of prostate cancer patients, showing its role in immune evasion [321]. Figure 5 demonstrates how lncRNAs participate in regulating therapy response and immune system in prostate cancer with an emphasis on molecular pathways.

### Exosomal lncRNAs

Recently, special attention has been directed towards extracellular vesicles (EVs) obtained from cancer and non-cancer cells [322, 323]. Overall, there are three main categories of EVs including exosomes, microvesicles and apoptotic bodies with functional roles in physiological and pathological conditions [324–326]. As nano-extracellular vesicles, exosomes are present in TME and various body fluids such as blood, saliva, pancreatic duct fluid, and amniotic fluid can participate in their transportation to distant tissues and organs [327]. Furthermore, they also function via autocrine and paracrine fluids [328]. Exosomes provide the communication among various cells and they contain various macromolecules such as proteins, lipids and most importantly, nucleic acids [329]. The exosomes originate from endosomal processing [330] and it has been reported that they contain ncRNAs, especially lncRNAs . Therefore, it is vital to reveal role of exosomal lncRNAs in cancer and in this section, we provide a description of exosome-mediated lncRNA delivery in prostate cancer and its association with malignant behavior [331].

It is worth mentioning that exosomal lncRNAs can be utilized for distinguishing prostate cancer and BPH. A clinical study collected urine samples from 30 prostate cancer patients and 49 BPH patients to examine potential of lncRNAs GAS5 and lincRNA-p21 in prostate cancer diagnosis. The expression level of exosomal GAS5 demonstrates no difference among prostate cancer and BPH. However, exosomal lincRNA-p21 lncRNA was different among patients with prostate cancer and BPH with more expression level in prostate cancer [332]. Another experiment investigated expression level of two exosomal lncRNAs including SAP30L-AS1 and SChLAP1 in prostate cancer and BPH. The results reveal high expression of exosomal lncRNA SAP30L-AS1 in BPH, while SChLAP1 shows more expression in prostate cancer compared to BPH [333]. Therefore, by developing novel imaging methods for tracing exosomes such as Antares2-mediated bioluminescence resonance energy transfer (BRET), a revolution can be made in cancer diagnosis [334].

LncRNAs are potent modulators of different molecular pathways in prostate cancer and microRNAs (miRNAs) are among the most common downstream targets of lncRNAs [335]. An interesting experiment has revealed that certain lncRNAs are enriched in prostate cancer exosomes and lncRNAs regulating miRNA expression are among them. Exosomal lncRNAs ELAVL1 and RBMX are enriched in prostate cancer due to their capacity in regulating expression level of miRNAs such as miRNA-17, miRNA-18a, miRNA-20a, miRNA-93 and miRNA-106b [336]. In fact, exosomes accelerate transfer of lncRNAs into extracellular milieu and based on the role of lncRNA as tumor-suppressor or tumor-promoting factor, it affects proliferation and invasion of prostate cancer cells [337]. Although a few studies have evaluated role of exosomal lncRNAs in prostate cancer, it appears that these kinds of lncRNAs can be considered as novel diagnostic and prognostic factors in prostate cancer and their expression level is of importance for distinguishing among BPH and prostate cancer. Furthermore, more diagnostic tools should be developed for detecting exosomes in prostate cancer. Table 2

**Table 2:** An overview of lncRNAs involved in prostate cancer progression/inhibition

LncRNA	Signaling network	Remarks	Refs
UNC5B-AS1	Caspase-9	Enhanced expression of UNC5B in prostate cancer cells and tissues Negative association between UNC5B-AS1 and caspase-9 and presence of negative feedback loop Preventing apoptosis	[338]
GASL1	GLUT1	Significant difference in GASL1 expression in normal and prostate cancer tissues Reducing GLUT1 expression and increasing Bcl-2 expression Overexpression of GASL1 suppresses tumor growth and invasion	[339]
GAS5	-	Exposing prostate cancer cells to dexamethasone enhances expression level of GAS5 to suppress proliferation and stimulate cell cycle arrest	[340]
EMX2OS	cGMP/PKG	Low expression of EMX2OS in prostate cancer, revealing its tumor-suppressor role Overexpression of EMX2OS and TCF12 jointly induces cGMP/PKG pathway to inhibit growth and viability of cancer cells	[341]
UCA1	MDM2/E-cadherin	Preventing the interaction between MDM2 and E-cadherin Increasing stability of E-cadherin Preventing aggressive behavior of prostate cancer cells	[342]
TMPO-AS1	-	This lncRNA can be considered as a prognostic and diagnostic tool in prostate cancer Overexpression of TMPO-AS1 is associated with undesirable prognosis Apoptosis inhibition	[343]
NCK1-AS1	-	Overexpression of NCK1-AS1 in prostate cancer and can be utilized for distinguishing with BPH patients	[344]
NR2F2-AS1	CDK4	Acting as tumor-promoting factor Increasing expression level of CDK4 Mediating cell cycle progression	[345]
GAS5	-	Association of GAS5 with translational elongation, protein biosynthesis and transcription Apoptosis inhibition Increasing proliferation and cell cycle progression	[346]
SOCS2-AS1	TNFSF10	Upregulation of SOCS2-AS1 in prostate cancer Down-regulation of TNFSF10 by lncRNA Apoptosis inhibition	[347]
HOTAIR	-	HOTAIR can drive neuroendocrine differentiation of prostate cancer	[348]
POTEF-AS1	-	Increasing growth and cell cycle progression Inhibiting apoptosis via down-regulating TLR pathway Mediating docetaxel resistance via suppressing apoptosis	[349]
MIR4435-2HG	FAK/Akt/β-catenin	Overexpression of MIR4435-2HG in prostate cancer cells and tissues Silencing this lncRNA prevents proliferation and invasion MIR4435-2HG cooperates with ST8SIA1 to induce FAK/Akt/β-catenin signaling, leading to prostate cancer progression	[350]
SNHG1	hnRNPL/EMT	Interaction of SNHG1 with hnRNPL to induce EMT in prostate cancer via E-cadherin down-regulation and vimentin upregulation Increasing migration and metastasis of cancer cells	[351]
ARLNC1	AR	Upregulation of ARLNC1 by AR ARLNC1 can also promote AR stabilization via RNA-RNA interaction Increasing prostate cancer progression	[352]
LBCS	hnRNPK/AR	Interaction of LBCS with hnRNPK to suppress AR translation Low expression of LBCS is associated with poor prognosis Preventing castration resistance in prostate cancer	[353]

### Therapeutic targeting of lncRNAs

As lncRNAs are considered as critical regulators of molecular pathways and mechanisms in prostate cancer, it is of importance to regulate their expression level to affect progression of prostate cancer cells. As it was discussed, most of the experiments have focused on revealing role of tumor-promoting lncRNAs in prostate cancer. Therefore, decreasing expression of such lncRNAs can pave the way to effective treatment of prostate cancer. In this section, our aim is to show currently applied therapeutic strategies in regulating expression levels of lncRNAs in prostate cancer.

#### Genetic intervention

RNA interference (RNAi) was first discovered in 1998 and it is a biological mechanism occurring in most eukaryotic cells, when double-stranded RNA (dsRNA) induces biochemical events. RNAi leads to sequence-specific inhibition of target gene expression [354]. The first clinical application of RNAi was in 2004, when a naked siRNA, called Bevasiranib was utilized for topical intravitreal injection for treatment of age-related diseases [355]. siRNA and short-hairpin RNA (shRNA) are among the most common genetic tools applied in disease therapy. shRNA is a potent genetic tool applied in basic research and genome engineering, while siRNA has opened its way in clinical course [356]. siRNA is considered as a synthetic short non-coding RNA that is inactive in cells until it is loaded into Argonaute (Ago2) via RNA-binding protein (TRBP). Then, passenger or sense stranded is eliminated, while guide or antisense stranded remains attached to catalytic Ago2. At the next step, guide strand of siRNA binds to seed region of messenger RNA (mRNA) and then, Ago2 cleaves it, resulting in expression suppression [357–360]. However, siRNA has a variety of impediments before targeting genes and reducing their expression level. It has been reported that siRNA can be degraded by endogenous ribonuclease enzymes in plasma, and it can undergo clearance by kidney filtration. Furthermore, siRNA should effectively penetrate into cancer cells and escape endosome-mediated degradation [361]. In order to overcome such challenges, nanocarriers have been developed for targeted delivery of siRNA into cancer cells, protecting against RNase degradation, and mediating endosomal escape [52, 362–364]. Noteworthy, siRNA can be applied for downregulating lncRNA expression in cancer therapy, and subsequent inhibition of proliferation and migration of cancer cells [365, 366].

The newly conducted experiments have exploited siRNA in affecting lncRNA expression in prostate cancer therapy. The expression level of lncRNA MNX1-AS1 undergoes upregulation in prostate cancer cells and tissues to mediate their growth and metastasis. Silencing lncRNA MNX1-AS1 by siRNA is correlated with suppressing prostate cancer migration via reducing N-cadherin and vimentin levels and increasing E-cadherin levels [367]. Besides, potential of prostate cancer cells in colony formation and proliferation can be suppressed using siRNA for lncRNA down-regulation [368]. Using siRNA for targeting lncRNAs can affect downstream molecular pathways involved in prostate cancer progression. LncRNA plasmacytoma variant translocation 1 (PVT1) is a tumor-promoting factor located on chromosome 8q24 adjacent to MYC [369]. In prostate cancer, lncRNA PVT1 induces phosphorylation of p38 to promote both proliferation and invasion. Silencing PVT1 using siRNA is associated with a significant decrease in survival and invasion of prostate cancer cells via preventing p38 phosphorylation [370]. It is worth mentioning that siRNA is beneficial in revealing role of lncRNAs in prostate cancer. For instance, lncRNA GAS5 is a tumor-suppressor factor in prostate cancer and its overexpression decreases miRNA-103 to inhibit Akt/mTOR signaling, leading to a significant decrease in proliferation and metastasis. In this case, siRNA application diminishes GAS5 expression in increasing prostate cancer progression, revealing anti-tumor activity of GAS5 [371].

The potential involvement of lncRNAs in drug resistance feature of prostate cancer cells has made them as ideal candidates for therapeutic targeting. Recently, we have shown that lncRNA HORAS5 overexpression triggers resistance of CRPC cells to taxane chemotherapy. This is mediated via upregulation of BCL2A1 that induces resistance of cancer cells to chemotherapy-mediated apoptosis. Silencing lncRNA HORAS5 via siRNA significantly reduces IC_50_ of cabazitaxel, enhancing efficacy of chemotherapy in prostate cancer therapy [372]. Although studies have clearly showed role of siRNA in reducing expression level of tumor-promoting lncRNAs and suppressing prostate cancer progression [220], there are some limitations that should be addressed. As it was mentioned, siRNA delivery is a vital requirement due to protecting against degradation and providing targeted delivery. However, experiments have just focused on using siRNA for downregulating lncRNAs in prostate cancer therapy. Therefore, future experiments can focus on using nanoarchitectures for siRNA delivery in prostate cancer therapy. Another limitation is that experiments have just used siRNA for lncRNA regulation. There are other genetic tools such as shRNA and CRISPR/Cas9 that their potential in lncRNA expression modulation should be explored.

#### Pharmacological intervention

**Fig. 6:**
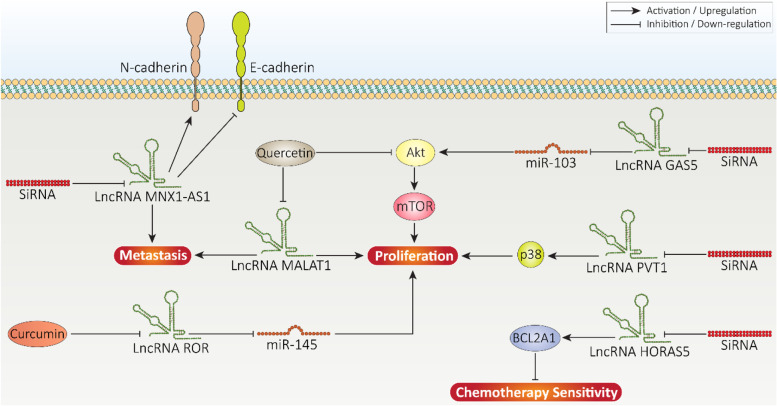
Therapeutic targeting of lncRNAs in prostate cancer. Anti-tumor compounds such as curcumin and siRNA can be used to decrease expression level of tumor-promoting lncRNAs in impairing progression of prostate cancer cells

In addition to genetic tools, anti-tumor compounds can also be utilized for targeting lncRNAs in prostate cancer. However, anti-tumor compounds targeting lncRNAs are mostly phytochemicals and suffer from poor bioavailability and for introducing them to clinic, strategies such as application of drug delivery systems should be considered to improve their potency [373]. Quercetin is a plant derived-natural compound that is extensively applied in prostate cancer therapy. Quercetin can suppress proliferation and migration of prostate cancer cells, and significantly enhances their response to chemotherapy. Furthermore, in order to improve anti-tumor activity of quercetin against prostate cancer, nanoparticles have been developed for its delivery [374]. LncRNAs are targets of quercetin in prostate cancer therapy. In this way, quercetin down-regulates expression level of MALAT1 in a concentration- and time-dependent manner. In addition to *in vitro* experiment, *in vivo* experiment on xenograft tumors has shown role of quercetin in suppressing prostate cancer progression. By downregulating lncRNA MALAT1, quercetin inhibits metastasis via EMT suppression. Furthermore, quercetin inhibits PI3K/Akt pathway to suppress proliferation [375]. Curcumin is another well-known anti-tumor agent, isolated from rhizome and root of *Curcuma longa* that can suppress prostate cancer progression via inducing apoptosis and cell cycle arrest, down-regulating NF-κB signaling and inhibiting angiogenesis [376]. Curcumin administration negatively affects prostate cancer stem cells and suppresses their growth and migration. LncRNA ROR functions as ceRNA to reduce miRNA-145, leading to prostate cancer progression. Curcumin administration reduces ROR expression, while it promotes miRNA-145 expression to effectively suppress prostate cancer progression [377]. **Figure** **6** depicts a summary of genetic and pharmacological interventions for regulating lncRNA expression in prostate cancer.

### Biomarker role and clinical application

With respect to high incidence rate and death resulting from prostate cancer, it is vital to translate pre-clinical findings to clinic for treatment of prostate cancer patients. LncRNAs can be considered as prognostic and diagnostic tools in prostate cancer. LncRNA ATB is a tumor-promoting factor capable of promoting both growth and invasion (EMT) of prostate cancer cells. The overexpression of lncRNA ATB is correlated with undesirable prognosis in prostate cancer patients [378]. As lncRNAs can affect immune system in providing immune evasion of prostate cancer cells, their expression level can determine response to immunotherapy [379]. In contrast to tumor-promoting lncRNAs that demonstrate high expression in prostate cancer, tumor-suppressor lncRNAs undergo significant down-regulation. It has been reported that lncRNA TINCR has close association with clinical T stage, lymph node and distant metastasis in prostate cancer. The expression level of TINCR is important in clinical course that its low expression shows poor prognosis [380]. The downregulation of tumor-suppressor lncRNAs such as DGCR5 reduces survival of prostate cancer patients [304]. Therefore, identification of these lncRNAs and investigating their expression level can be utilized as a reliable and potent prognostic tool [381]. Furthermore, it was discussed in previous section that expression level of exosomal lncRNAs can be examined in serum of prostate cancer patients as diagnostic and prognostic tools [332].

### Conclusion and remarks

The present review article investigated role of lncRNAs in prostate cancer [382–385]. The expression level of lncRNAs is different among prostate cancer patients and BPH patients, so they can be considered as reliable biomarkers. LncRNAs are capable of regulating proliferation and metastasis of prostate cancer cells. Furthermore, autophagy and apoptosis as two major arms of programmed cell death, are modulated by lncRNAs in prostate cancer. A variety of downstream targets of lncRNAs have been identified that among them, STAT3, NF-κB, PTEN, PI3K/Akt and miRNAs are the most important ones. The tumor-promoting lncRNAs demonstrate an increase in expression in prostate cancer, while expression level of tumor-suppressor lncRNAs undergoes down-regulation. In addition to proliferation and migration, lncRNAs can regulate response of prostate cancer cells to chemotherapy and radiotherapy. Based on pre-clinical studies, lncRNAs induce resistance to PTX and DOX chemotherapy. Therefore, for providing effective cancer chemotherapy, lncRNAs involved in DOX and PTX resistance should be suppressed. Furthermore, lncRNAs can inhibit autophagy in mediating radio-resistance. However, lncRNA and autophagy interaction should be evaluated with more details due to pro-survival and pro-death functions of autophagy in prostate cancer.

To suppress prostate cancer progression, anti-tumor immunity is activated, and cytotoxic T cells are vital for this purpose. However, lncRNAs can induce PD-1 expression in preventing proliferation of cytotoxic T cells and mediating their apoptosis, leading to immune evasion of prostate cancer. Therefore, for effective immunotherapy, it is necessary to identify such lncRNAs to improve potential of immunotherapy. In respect to vital role of lncRNAs, pharmacological and genetic interventions have been performed to target lncRNAs in favor of prostate cancer suppression. For clinical course, lncRNAs can be utilized as diagnostic and prognostic tools for prostate cancer patients. Future experiments can focus on discovering more lncRNAs involved in prostate cancer progression/inhibition to pave the way for treatment of this malignant condition.

## Data Availability

Not applicable

## Abbreviations

BPH: Benign prostatic hyperplasia
PSA: Prostate specific antigen
CRPC: Castration-resistant prostate cancer
AR: Androgen receptor
DDR: DNA damage repair
ADT: Androgen-deprivation therapy
STAT3: Signal transducer and activator of transcription 3
Hh: Hedgehog
PTEN: Phosphatase and tensin homolog
PI3K: Phosphatidylinositol 3-kinase
Akt: Protein kinase-B
NF-kB: Nuclear factor-kappaB
ncRNAs: Non-coding RNAs
lncRNAs: Long non-coding RNAs
siRNA: Small interfering RNA
piRNA: Piwi-interacting RNA
ORF: Open reading frame
UTRs: Untranslated regions
ceRNA: Competing endogenous RNA
5^/^-UTR: 5^/^-Untranslated region
mRNA: Messenger RNA
CCAT1: Colon cancer associated transcript-1
ER α: Estrogen receptor- α
FOXP4: Forkhead box P4 antisense RNA 1
PAX5: Paired box 5
TGF-β: Transforming growth factor-beta
Fz: Frizzled
LRP: Low-density lipoprotein receptor-related protein
GSK-3 β: Glycogen synthase kinase-3beta
NORAD: Noncoding RNA activated by DNA damage
AIPC: Androgen-independent prostate cancer
JAK: Janus kinase
NED: Neuroendocrine differentiation
En: Enzalutamide
EZH2: Enhancer of zeste homolog 2
PIP3: Phosphatidylinositol-3,4,5-phosphate
mTOR: Mammalian target of rapamycin
5-FU: 5-flourouracil
LASP1: LIM and SH3 protein 1
TNF- α: tumor necrosis factor-α
IL-1: Interleukin-1
IKK: IκB kinase
CHRF: Cardiac hypertrophy-related factor
EMT: Epithelial-to-mesenchymal transition
PHLPP: PH and leucine-rich repeat protein phosphatase
GLUT-1: Glucose transporter-1
TLR: Toll-like receptor
PCAT7: Prostate cancer-associated transcript 7
CXCR-4: C-X-C chemokine receptor type 4
FGF2: Fibroblast growth factor 2
WDR5: WD repeat domain 5
DOX: Doxorubicin
RORγ: Retinoic acid-related orphan nuclear receptor γ
EGFR: Epidermal growth factor receptor
PTX: Paclitaxel
FSCN1: Fascin1
AMPK: AMP-activated protein kinase
HULC: Highly upregulated in liver cancer
NK: Natural killer
CTLs: Cytotoxic T cells
DKK1: Dickkopf-1
PD-1: Programmed death-1
PD-L1: Programmed death-ligand 1
Tcm: T central memory
pDCs: Plasmacytoid dendritic cells
EVs: Extracellular vesicles
BRET: Bioluminescence resonance energy transfer
miRNAs: microRNAs
RNAi: RNA interference
dsRNA: Double-stranded RNA
shRNA: Short-hairpin RNA
Ago2: Argonaute 2
PVT1: Plasmacytoma variant translocation 1
